# All-Polymer Multilayer Lab-on-Fiber Ultrasonic Detectors in the Biomedical Field: A Numerical Study in Pursuit of Photoacoustic Applications

**DOI:** 10.3390/s25237349

**Published:** 2025-12-02

**Authors:** Barbara Rossi, Maria Alessandra Cutolo, Paolo Massimo Aiello, Giovanni Breglio, Andrea Cusano, Martino Giaquinto

**Affiliations:** 1 Department of Electrical Engineering and Information Technology, University of Naples Federico II, 80125 Naples, Italy; 2 Department of Engineering, University of Sannio, 82100 Benevento, Italy; 3 Department of Information Engineering, Electrical Engineering and Applied Mathematics, University of Salerno, 84084 Fisciano, Italy

**Keywords:** lab-on-fiber technology, fiber-optic sensors, fiber-optics

## Abstract

The development of minimally invasive diagnostic devices in the biomedical field has grown significantly, especially those that take advantage of photoacoustic phenomena. Photoacoustic imaging is an imaging technique that exploits the photoacoustic effect, relying on the conversion of absorbed light into ultrasound waves. Thanks to lab-on-fiber technology, optical fiber can be functionalized to generate and receive a photoacoustic signal. Weak acoustic signals often limit this process, as conversion efficiency can be influenced by factors such as tissue heterogeneity, light scattering, and the attenuation of the acoustic waves within tissues. Consequently, there is significant interest in the development of highly sensitive systems with broad bandwidths. While the literature has largely focused on standard devices utilizing the interferometric effect in homogeneous slabs, this study explores the potential of multilayer structures that leverage Bragg reflection to be realized on the fiber tip. We numerically investigated both periodic and aperiodic designs of polymeric multilayer structures to further enhance the optical performance of opto-acoustic sensors. We demonstrate an enhancement in sensitivity of up to about three orders of magnitude without compromising bandwidth. This work highlights the advantages of multilayer sensor designs in improving sensitivity and performance for high-frequency opto-acoustic sensing.

## 1. Introduction

In recent years, there has been growing interest in all-optical ultrasound transducers [1]. These systems generally consist of two specifically engineered optical fibres, one for generation and one for detection of ultrasound waves, featuring biocompatibility, lack of electrical connections, and compact size [2]. Ultrasound waves are generated via the photoacoustic effect: pulsed laser light is absorbed by the target, producing rapid heating, thermoelastic expansion, and the emission of acoustic waves. However, the resulting signals are often weak and further attenuated by tissue heterogeneity and scattering, making detection challenging. Ultrasound detection is commonly achieved using interferometric structures based on homogeneous thin films deposited at the fiber tip. These sensors inherently face a trade-off between sensitivity and bandwidth, which places a fundamental limitation on their design and application [3]. This aspect is particularly significant in high-resolution imaging applications, where operating within frequency bands of tens of MHz is essential to achieve the desired spatial resolution [4]. At higher frequencies, sensitivity tends to decrease, making the detection of weak signals more challenging. Therefore, there is a strong interest in developing sensors that combine high sensitivity with broad bandwidth [5,6]. In this context, Beard et al. demonstrated the photoacoustic imaging capabilities of a plano-concave Fabry–Pérot (FP) fiber-optic transducer [7]. This state-of-the-art solution enables beam confinement to enhance optical sensitivity. It offers an omnidirectional response up to tens of megahertz, addressing challenges typical of FP interferometers. Typically fabricated via dip-coating, these structures have limited process control, with sensor curvature influenced by fiber dimensions and surface tension. In a recent study, we numerically investigated the effects of curvature, focusing on its performance and tolerance to fabrication [8]. Specifically, a curved structure with a curvature that matches the light beam was investigated, a design that can be fabricated using the advanced technique of two-photon lithography (TPL). Our findings show that a curved surface is robust against fabrication variations, enabling it to closely approximate the performance of an idealized structure. However, for thicknesses below the Rayleigh zone (around 45 µm), this design does not yield significant improvements in sensitivity or bandwidth. Consequently, we shifted our focus to multilayer structures based on Bragg reflection, which enable acoustic sensing through frequency-selective resonance shifts induced by acoustic waves. These configurations offer a greater number of degrees of freedom in both mechanical and optical properties, which can be strategically exploited to enhance the overall optical performance of the system. Fiber Bragg gratings (FBGs) are widely used in sensing applications [9]. However, traditional FBGs are typically inscribed within the fiber core, and their performance is inherently limited by the material properties of the fiber itself. On the other hand, polymeric materials are particularly well suited for acoustic wave detection due to their flexibility, mechanical properties, and ease of processing. Leveraging advances in lab-on-fiber technology [10] and recent fabrication techniques, the idea of realizing a fully polymeric multilayer structure directly on the fiber tip has emerged. This approach enables the design of sensors capable of detecting ultrasonic signals with enhanced flexibility and tailored mechanical properties, independent from the intrinsic constraints of the fiber material. Other studies have explored the use of multilayer structures in the context of acoustic sensing. For example, Jeong et al. in their work [11] presented a flexible composite sensor with enhanced sensing performance under varying pressure and temperature conditions, achieved by engineering the composite’s structure, to tailor its mechanical properties. So far, opto-acoustic multilayer structures have largely been developed using non-polymeric materials [12] or polymeric materials alternating with metals. Yang et al. [13] propose multilayer polymer–metal structures for acoustic impedance matching in the design of high-frequency broadband ultrasonic transducers. The alternating polymer–metal structure requires a piezoelectric element and results in a bulky design. Polymeric multilayer structures have been realized by Alvarez et al. [13], who investigated the optical behavior of polymeric multilayers. The results confirmed precise layer control, highlighting the feasibility and reliability of thick layer stacks. Despite these advances, the application of polymer multilayer structures directly on optical fibers is still relatively uncommon. Recently, Kim et al. [14] demonstrated an innovative multilayer all-polymer metasurface directly stacked on an optical fiber, showcasing a compact and versatile approach for integrating polymer multilayer structures with fiber-optics.

In this study, we explore the potential of an all-polymeric structure on the fiber tip for acoustic detection in biomedical field. This work represents a preliminary numerical analysis aimed at developing sensors suitable for photoacoustic imaging applications. Accordingly, the design process incorporates application-specific constraints, such as the requirement for broadband frequency response extending into the tens of MHz range and sensitivity sufficient to detect pressure levels on the order of kPa [15]. In this scenario, we propose both periodic and aperiodic configurations, hereafter referred to as thickness-matched, optical path-matched, and π-shifted designs. This allows us to understand how structural symmetry and its disruption affect the sensor acoustic and optical response. The analysis was conducted by starting with well-established equations to form the foundation of the design. Then the results were validated through a Finite Element Method (FEM) approach, enabling a detailed numerical analysis of the structure’s performance in terms of sensitivity and resonance frequency and bandwidth. Finally, we compared the multilayer structure with a conventional structure (FP) to evaluate their potential for high-frequency opto-acoustic sensing applications.

The manuscript is structured to first present the model and evaluation metrics (Section 2), followed by the presentation of results and their discussion. In particular, the Results section includes separate analyses of the periodic (Section 3.2.1) and aperiodic structures (Section 3.2.2) and concludes with a comparative discussion highlighting their relative advantages.

## 2. Materials and Methods

In this section, we present the multilayer structures given by the alternation of two different polymers under investigation. In this study, we investigate the effect of the layer thicknesses, sensor length, and their combined influence on the Bragg wavelength, the sensor sensitivity, and the detection bandwidth in order to identify the optimal layer dimensions that allow the sensor to operate within the standard optical fiber range while maintaining high sensitivity and a wide bandwidth. To this end, we analyze two types of structures; these are a periodic configuration obtained under two different conditions (thickness-matched and optical path-matched) and a π-shifted (aperiodic) structure, which introduces a central phase shift to investigate its effect on overall performance. The models and evaluation metrics adopted to quantify the performance of the proposed structure are defined in Section 2.2 and Section 2.3.

### 2.1. Analyzed Structure

**Figure 1:**
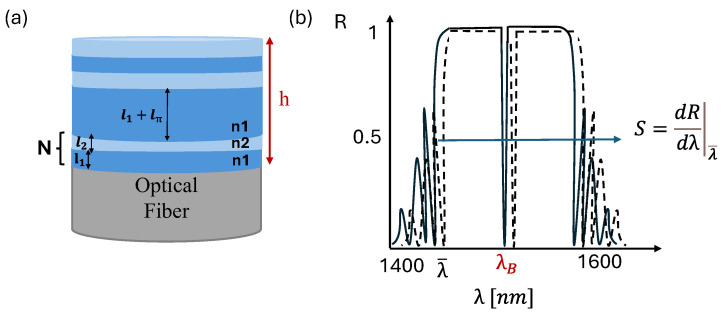
(**a**) Two-dimensional schematic of the multilayer aperiodic structure; (**b**) example of expected reflection spectra of the multilayer structure in the range of interest.

The multilayer structure objective of this analysis consists of consecutive bilayer polymer pairs with thicknesses and refractive indices denoted *l*_1_ and *l*_2_, *n*_1_ and *n*_2,_ respectively (Figure 1a). Here, $l_{\pi }$ represents a possible central asymmetry layer thickness. The total structure length *h* is given by the repetition of *N* bilayers plus the asymmetry layer, as described by the following formula:

*h* = *N* ∙ (*l*_1_ + *l*_2_) + *l_π_* (1)

Note that Figure 1a represents the most general case, which also allows for the introduction of a central asymmetry $l_{\pi }$, generating a π-Bragg structure. However, for simplicity of treatment, the numerical equations presented here assume a symmetric structure with $l_{\pi }=0$, and therefore this term is not included in the equations that follow. Upon the interaction of an incident acoustic wave with the multilayer sensor, the wave induces deformations in the layers, leading to variations in their thicknesses.

The way in which deformation occurs can be described using Hooke’s law, which models the relationship between stress and strain in elastic materials. Essentially, the displacement (∆h) of a material depends on the stress (*σ*) and its stiffness, represented by the Young’s modulus (*E*). Therefore, the displacement of each layer is given by the formula $∆h_{i}\;=\sigma_{i}\times l_{i}/E_{i}$, *i* = 1, 2, where *σ* is the applied stress, E is the Young’s modulus of the first material, l is the thickness of the layer, and *i* refers to the polymer considered. In our analysis we consider an incident pressure of $\sigma_{1}=\sigma_{2}=1\;kPa$, consistent with pressure levels typically generated in photoacoustic imaging experiments, which commonly range from hundreds of pascals to a few kilopascals [15]. The total displacement ∆*h* is given by the sum of individual displacements experienced by the structure and can be expressed as

(2) $$∆h=N\;\cdot \;(∆l_{1}+∆l_{2})$$

The displacement of the polymeric structure is frequency-dependent, exhibiting a flat response across a range of frequencies until it reaches resonance at a specific frequency, where it experiences maximum [3]. This resonance frequency is crucial for detection purposes, with a higher resonance frequency being desired to achieve a broader bandwidth. The resonance frequency $f_{0}$ of the structure can be estimated using the wave equation for a thin vibrating plate in its fundamental mode as a function of the material’s mechanical properties such as Young’s modulus and the density of the layers, as follows:

(3) $$f_{0}=\;\frac{1}{4h}\sqrt{\frac{E_{m}}{\rho_{m}}}$$

where $E_{m}$ is the effective Young’s modulus of the multilayer structure. When two layers with thicknesses $l_{1}$ and $l_{2}$ and Young’s moduli $E_{1}$ and $E_{2}$ are subjected to a uniaxial load perpendicular to the layers, the total deformation is the sum of the deformations of each layer. Assuming the same force acts through both layers, the effective Young’s modulus *E_m_* can be expressed using the following [16]:

(4) $$E_{m}=\;(l_{1}+l_{2})\cdot {\left(\frac{l_{1}}{E_{1}}+\frac{l_{2}}{E_{2}}\right)}^{-1}$$

while the average density $\rho_{m}$ is defined as

(5) $$\rho_{m}=\frac{l_{1}\cdot \rho_{1}+l_{2}\cdot \rho_{2}}{l_{1}+l_{2}}$$

where $\rho_{1}$ and $\rho_{2}$ are the densities of the single polymeric layer. Note that the resonance frequency can be fine-tuned by adjusting the thicknesses of the layers and the material properties, enabling the sensor response to be tailored to specific applications.

From an optical perspective, the multilayer structure presents a reflectivity spectrum that is centered according to the relationship between the Bragg central wavelength and the layer thicknesses *l*_1_ and *l*_2_ [17]:

(6) $$M\cdot \lambda_{B}=2\;\cdot \;\left({n_{1}\cdot \;l}_{1}+n_{2}\cdot l_{2}\right)$$

where $n_{1}$ and $n_{2}$ are the refractive index of the polymer and *M* is an integer number indicating the order of refraction. In our analysis, we focus only on first-order phenomena, thus assuming *M* = 1. According to Equation (6), a variation in the layer thickness leads to a shifts in the interference spectrum that can be detected (Figure 1b). In this work, the shifted spectrum is obtained by considering the layer-formed structure, where each layer’s thickness is updated according to the displacement calculation outlined above (Equation (2)). We also studied the effect of a symmetry break introducing a variation $l_{\pi }$ in the central layer thickness without changing *h*, which causes an artifact in the spectrum (Figure 1b). In principle, the sensor is illuminated with a focused continuous-wave laser beam at a working wavelength $\lambda^{*}$ tuned to the edge of the interference (Figure 1b). Under these conditions, the stress due to an incident acoustic wave modulates the optical path producing a corresponding modulation in the reflected optical power. The fiber sensors can be directly coupled to the interrogation laser and photodiode via an optical circulator, exploiting a component able to work in the tens of MHz range, as required for high-frequency photoacoustic imaging. This approach was shown in previous works such as that of Guggenheim et al. [7] and Ansari et al. [1], where similar configurations were successfully adopted. To detect high-frequency signals, many ultrasound-based sensors operate in the linear region of interference fringe, ensuring a linear transfer function, no ambiguity in the fringe direction, simple signal processing, and the highest sensitivity at the quadrature point [3,18]. The quadrature point is susceptible to drift due to ambient interferences and source variation, causing signal attenuation. In practice, the phase shift is induced by quasi-static loading or an external temperature change occurring at low frequencies due to the slow nature of the mechanical or thermal stimuli [19]. In contrast, acoustic signals induce comparatively small phase shifts but at significantly higher frequencies. For this reason, a feedback control system can be employed to track and compensate for the slow, large-scale phase drift by maintaining the operating point at the quadrature point of the interferometer. This method, which relies on modulating the amplitude of the reflected beam to generate a signal correlated with the acoustic wave amplitude, is more effective than demodulating the wavelength shift of a spectral feature (such as a spectral minimum or maximum), as the latter requires extracting spectral features at a rate that matches the acoustic frequency.

In our analysis, we focus on biocompatible polymeric materials due to their suitability for medical applications. Increasing the refractive index gap leads to a higher reflectivity value and wide reflection spectrum, essential for improving performance. With polymeric materials, a refractive index contrast of at most ~0.3 can be achieved [20]. For these reasons, we consider polymer 1 and polymer 2 to have a refractive index of *n* = 1.7 and *n* = 1.4, respectively, along with Young’s moduli of 3.2 GPa and 2.5 GPa and densities of 1.1 $g/{cm}^{3}$ and 1.7 $g/{cm}^{3}$ as their mechanical properties [21,22,23,24]. Representative polymer pairs exhibiting a similar refractive index contrast include polystyrene/poly(vinylidene fluoride) (PS/PVDF) and polystyrene/poly(methyl methacrylate) (PS/PMMA). Indeed, recent approaches have shown that polystyrene (PS), thanks to pigment loading or molecular doping, can reach refractive indices up to *n* ≈ 1.7, enabling a larger index contrast and consequently enhancing the Bragg reflection response [22]. In this work, PS/PVDF is adopted as a representative system. The bi-layer thickness has been chosen to set reflection interferometric peaks at $\lambda_{B}=1.5$ µm, ensuring reflection spectrum within the 1.4–1.6 µm range, which is typical for basic optical telecom instruments and corresponds to the region of lowest loss in the spectrum of a single-mode optical fiber. Additionally, the thicknesses of *l*_1_ and *l*_2_ are constrained by fabrication limits, as the layers can be deposited using spin coating [25]. Specifically, considering resolution, defined as the minimum thickness increment that can be reliably controlled during fabrication, layer thickness can be adjusted and reproduced with precision down to the order of tens of nanometres [26]. As such, reported thickness values have been rounded to reflect the resolution limit. Tolerance, on the other hand, refers to the inherent variability introduced during the actual fabrication process, due to factors such as polymer viscosity, spin speed, and environmental conditions. This practical variability, estimated to be around 5% [27,28], influences the reproducibility and consistency of the resulting structures. Accordingly, Section 3.3 explores the influence of this tolerance on overall performance through a dedicated analysis.

### 2.2. Numerical Models

The optical response of the multilayer was studied using a function of the “Electromagnetic Waves and Antennas” toolbox of Matlab^®^ (version R2024b) named multidiel. The function multidiel—given the refractive indices of the materials $n_{1}$ and $n_{2}$, their thicknesses $l_{1}$ and $l_{2}$_,_ and periodicity N, along with the wavelength of the incident light and the angle of incidence—calculates the reflection coefficient (Γ) of a multilayered structure. It uses a recursive approach to account for multiple reflections and interference effects within the layers. The final output is the reflectance spectrum, R, obtained from R = |Γ|^2^, which represents the fraction of incident light that is reflected by the multilayer structure [27]. In our model, a plane electromagnetic wave with an angle of incidence of zero degrees propagates through the multilayer structure. The beam divergence is not considered, as the study was conducted within the Rayleigh region, where the beam can still be approximated as collimated, as investigated in a previous work [19]. In the optical analysis, the shift in the interference was determined by considering the displacement in layer thickness caused by the incident acoustic wave.

**Figure 2:**
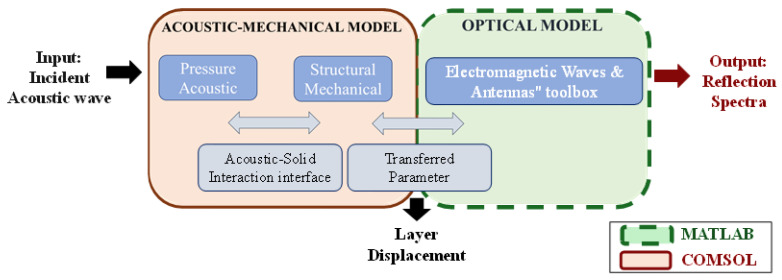
Diagram of the FEM models implemented in COMSOL Multiphysics, illustrating the coupling between the acoustic–mechanical and optical simulations. The acoustic–mechanical model, solved using the pressure acoustics and structural mechanics physics interfaces coupled through the acoustic–solid interaction module, simulates the structural response to an incident acoustic wave. The intermediate output of this model provides the layer displacement (compression), which is then used as input for the optical simulation, considering both the unperturbed and perturbed configurations. The optical model, developed using the electromagnetic waves & antennas toolbox in MATLAB, computes the reflection spectra and corresponding optical sensitivity. The overall sensor sensitivity is finally obtained by combining the acoustic and optical contributions.

The mechanical response was studied with an FEM approach using COMSOL Multiphysics software [28]. The proposed model simulates the dynamic behaviour of the multilayer system, considering the interaction between the mechanical properties of the materials and the incident acoustic waves at varying frequencies. Following methodologies presented in other studies [29], the problem is approached using an acoustic–mechanical model that employs two physics interfaces: pressure acoustics and structural mechanics. The propagation of the acoustic wave along the longitudinal direction of the model is essentially ruled by the Helmholtz equation. In this study, the interaction between the acoustic field and the sensor was modeled assuming an elastic medium representative of soft biological tissue. Considering that the active area of the sensor is significantly smaller than the acoustic wavelength at the reference frequency of 10 MHz, the incident acoustic waves were approximated as locally planar. This assumption is widely accepted in preliminary analyses and simplifies the modeling without compromising the accuracy necessary to evaluate the sensor’s frequency-dependent response. Under the assumption that nonlinear effects in tissues can be neglected and that the amplitude of shear waves is much smaller than that of pressure waves, the Helmholtz equation can be applied to model acoustic wave propagation inside soft tissue. In line with this approximation, the tissue was treated as a fluid with a density of 1000 kg/m^3^ and a speed of sound of 1540 m/s, neglecting frequency-dependent attenuation effects [30,31]. To properly couple these physics, it is necessary to impose the continuity of normal displacement at the solid–fluid interfaces, ensure static equilibrium between the pressure and the stress normal to the solid boundaries, and enforce zero tangential stresses at the fluid boundaries [31]. The external domain was surrounded by water, acoustically approximating the soft tissues’ behaviour well. Impedance boundary conditions were applied at its edges to minimize artificial reflections and to simulate an infinite extended domain. A preliminary analysis of the external domains was made to strike a balance between minimizing their impact on the solution and optimizing computational performance. The polymeric structure was modelled as a linear elastic and isotropic material [31]. Regarding the mesh, a free tetrahedral mesh was utilized. In the surrounding water domain, a mesh number of elements n_mesh_ was determined by the frequency f of the acoustic wave, using the equation

n_mesh _ = 1500/f/10 (7)

where 1500 [m/s] represents the assumed speed of sound in water. Additionally, in the multilayer structure, a finer mesh was employed to accurately capture the mechanical behavior and interactions between the layers. The analysis was conducted in the frequency domain under the assumption of linear elasticity and steady-state harmonic excitation. As such, the reported thickness variations represent the amplitude of the periodic deformations experienced by the structure at each excitation frequency. In this context, the deformation can be interpreted as the steady-state solution of the acoustic–mechanical equations, and its magnitude reflects the frequency-dependent mechanical sensitivity of the sensor [31]. The values obtained from the COMSOL model were subsequently incorporated into the optical simulation to model the deformed structure and derive the final results. This approach ensures that the mechanical deformation, proportional to the applied pressure and dependent on the Young’s modulus of the polymer layers, is consistently transferred to the optical model, allowing a realistic evaluation of the sensor’s response. A schematic diagram of the FEM models implemented in COMSOL Multiphysics is shown in Figure 2, illustrating the coupling between the acoustic–mechanical and optical simulations. The input of the model is an incident acoustic wave that interacts with the polymeric structure. The intermediate output of this model provides the layer displacement (compression), which is then used as input for the optical simulation, considering both unperturbed and perturbed configurations. The optical model, developed using the Electromagnetic Waves and Antennas toolbox in MATLAB, computes the reflection spectra and corresponding optical sensitivity. The overall sensor sensitivity is finally obtained by combining the acoustic and optical contributions.

The results obtained with the multilayer configuration were compared with those from a benchmark structure, given by a planar FP cavity simulated under the same conditions with optical and mechanical properties of polymer 1 (Section 2.1).

### 2.3. Evaluation Metrics

High sensitivity and broad bandwidth are essential requirements for optical fiber-based acoustic sensors, as they directly influence the spatial resolution and frequency range over which the sensor can accurately detect acoustic signals [9]. The assessment of the opto-acoustic performance of the fiber-optic detectors is based primarily on two key metrics: sensitivity *S* and frequency $f_{0}$. These two parameters summarize the detector’s ability to transduce an acoustic pressure into a measurable optical signal and the frequency range over which this transduction is effective. Because the interaction with an incident acoustic wave causes geometric perturbations of the interferometric cavity that alter the optical spectrum, such as peak position, width, and shape, which are optical and acoustic (mechanical) parameters describing the spectral response and mechanical variations and must be introduced and evaluated. The following definitions and relations are adopted in this work.

For typical resonance-based optical ultrasound sensors, the sensitivity *S* can be defined as the reflectivity spectrum variation *dR* at an operative wavelength $\lambda^{*}$ as a function of an incident acoustic wave with pressure *P* at a fixed wavelength [8]. Indeed, *S* can also be expressed as a product of two factors, one related to the mechanical response, named acoustic sensitivity $S_{A},$ and one related to the optical behavior, named optical sensitivity, $S_{O}$, and expressed as follows:

(8) $$S={\left.\frac{dR}{dP}\right|}_{\lambda^{*}}={\left.\frac{dR}{dh}\right|}_{\lambda^{*}}\cdot \frac{dh}{dP}=S_{O}\cdot S_{A}$$

Here, $S_{A}$ describes the device length dh variation as a function of the acoustic wave amplitude *dP*. The acoustic sensitivity of primary interest here is the static value at the flat region of the frequency band, which we refer to as $S_{A,static}$. The optical sensitivity $S_{o}$ expresses the reflectivity spectrum variation *dR* at a given wavelength λ^*^ as a function of the length variation *dh*.

The working frequency of ultrasound sensors in terms of bandwidth and resonance frequency is crucial for image quality. Light-induced ultrasonic waves have a broad frequency spectrum ranging from kHz to hundreds of MHz. Thus, ultrasonic sensors should have a wide bandwidth and a high resonance frequency to improve image resolution. The resonance frequency (*f*_0_) is defined as the frequency at which sensitivity reaches its maximum, while the sensor bandwidth is defined as the frequency range up to the cut-off frequency *(f_cut-off_)*, i.e., the point at which amplitude is reduced by the −3 dB cut-off.

To test the robustness of the sensor against fabrication-induced geometrical variations, we performed a numerical tolerance analysis by introducing controlled thickness deviations and evaluating the resulting response through a compact metric based on the Figure of Merit (FOM). The FOM is defined as the product between the quality factor (Q-factor) and the visibility (V) of the interference peak. Since dimensional deviations can alter the shape of the peak, both the Q-factor and V and consequently the sensitivity may vary. To quantitatively assess the robustness of each configuration against such variations, we conducted a statistical analysis by introducing a random variation in the thickness of each layer, with values sampled within the tolerance of ±5%, and calculated the relative standard deviation of the FOM, $\sigma_{FOM}$ [%] [31]. This tolerance level is consistent with typical fabrication uncertainties, ensuring that the analysis realistically reflects practical manufacturing conditions [27]. The $\sigma_{FOM}$ was calculated across 100 random configurations. Lower $\sigma_{FOM}$ means that the results obtained from different configurations are tightly clustered, implying that the sensor response is more stable and less affected by perturbations in geometry. Therefore, a smaller $\sigma_{FOM}$ indicates higher robustness and greater consistency of sensor response under geometric perturbations.

## 3. Results and Discussion

This section presents the main findings of the study, focusing on the influence of the design parameters and the numerical validation of the proposed configurations. In particular, Section 3.1 analyzes the effect of the design parameters through a preliminary theoretical approach, while Section 3.2 reports the numerical results obtained for the proposed structures. The structures were investigated from both a mechanical and optical point of view. Finally, in Section 3.3, we summarize the main performance of the proposed structure and compare it in terms of sensitivity, resonance frequency and bandwidth. As part of the performance comparison, with a view toward a realistic implementation and testing of the proposed structures, we also report the dynamic operating range and sensitivity to fabrication-related thickness variations.

### 3.1. Preliminary Theoretical Considerations

**Figure 3:**
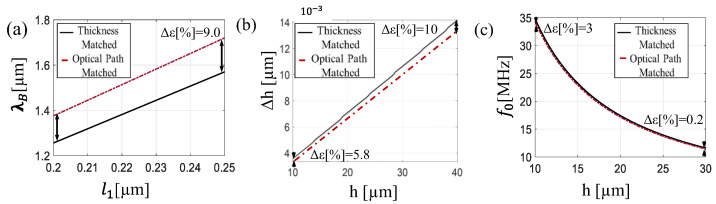
(**a**) Central resonance position *λ_B_* as a function of layer thickness *l*_1_ of thickness-matched solution (black solid line) and optical path-matched solution (red dash-dot line). (**b**) Total displacement Δ*h* as a function of sensor length h of thickness-matched solution (black solid line) and optical path-matched solution (red dash-dot line). (**c**) Resonance frequency $f_{0}$ as a function of sensor length *h* of thickness-matched solution (black solid line) and optical path-matched solution (red dash-dot line).

This section outlines the design strategy used to achieve an interference spectrum centered at a wavelength of *λ_B_* = 1.5 µm and systematically investigates the influence of key parameters. The analysis is based on the theoretical framework and equations introduced in Section 2.1, particularly Equations (2), (3), and (6), and the main design outcomes are summarized in Figure 3.

Two primary configurations are considered to fulfil the condition imposed by Equation (6): the thickness-matched configuration, where both layers have equal physical thicknesses ${(l}_{1}=l_{2})$, and the optical path-matched configuration, in which the optical paths are equivalent ($n_{1}\cdot l_{1}=\;n_{2}\cdot l_{2}$). Figure 3a shows the variation of the central wavelength as a function of *l*_1_, ranging from 0.20 µm to 0.25 µm and ensuring the satisfaction of the first-order interference condition (M = 1). The desired wavelength of *λ_B_* = 1.5 µm is achieved either when *l*_1_ = *l*_2_ = 0.24 µm, or when *l*_1_ = 0.218 µm and l2 =n1n2·l1=0.26 µm. The mechanical response of the sensor is then evaluated for both design solutions. As shown in Figure 3b, the total displacement Δ*h* increases linearly with the overall sensor thickness, resulting in enhanced sensitivity. However, increasing the sensor length also leads to a reduction in resonance frequency. Indeed, as shown in Figure 3c, the resonant frequency $f_{0}$ starting from 33 MHz at *h* = 10 µm arrives at 11 MHz at *h* = 30 µm. Based on these findings, a thickness of *h* = 30 µm was selected as a first validation point, maximizing sensitivity while maintaining a resonant frequency above 10 MHz, suitable for broadband photoacoustic applications.

### 3.2. Numerical Analysis

The following section presents a numerical validation of two different configurations, Periodic structure (Section 3.2.1) and π-shifted structure, when a symmetry break was introduced (Section 3.2.2). Firstly, we present the main results, i.e., the reflectivity spectrum and the sensitivity as a function of the frequency for each configuration, then, the optical and mechanical contributions to sensitivity are considered separately using the numerical model described in Section 2.2, allowing their individual impact to be examined.

#### 3.2.1. Periodic Structure

**Figure 4:**
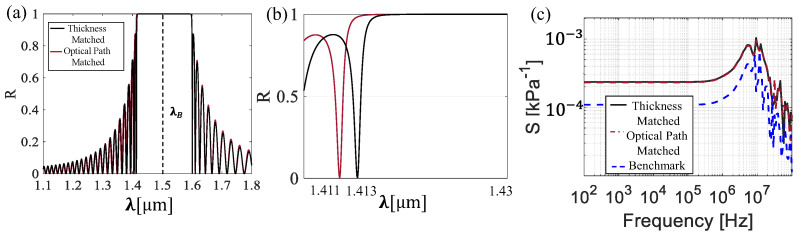
(**a**) Interference spectra of thickness-matched solution (black line) and optical path-matched solution (red line). (**b**) Zoom on the rising edge (left front) of the reflection spectrum being analyzed. (**c**) Sensitivity as a function of the frequency at *h* = 30 µm of the thickness-matched design (black solid line) and optical path-matched design (red line) and the benchmark configuration (blue dashed line).

Figure 4 illustrates the main outcomes of the numerical analysis, namely, the sensor reflection spectrum and its acoustic response at various frequencies. As shown in Figure 3a, the reflection spectrum of the sensor exhibits a broad spectral response, which is consistent with the significant difference in refractive indices between the layers. A central position of *λ_B_* = 1.5 µm is shown, with two distinct minima in reflectivity observed at wavelengths of 1.41 µm and 1.60 µm, corresponding to a total bandwidth of 0.19 µm. These results agree with the theoretical predictions, confirming that the central resonance is located around the wavelength *λ_B_*. To provide a more detailed view, Figure 3b zooms in on the region surrounding the first left null, where a reflectivity peak is formed between the high-reflectivity window and the first left-side lobe. This peak, occurring at a wavelength of approximately 1.413 µm and with a bandwidth of around 0.9 nm, was selected. Since sensitivity is calculated at a fixed wavelength, according to Equation (8), the operating wavelength $\bar{\lambda }$ is chosen as the wavelength at which the reflectivity reaches half of its maximum value located on the falling edge of the selected peak (Figure 4b). Then, the sensitivity of the two investigated designs, thickness-matched and optical path-matched, along with the benchmark structure, is shown in Figure 4c.

The structures exhibit a flat response at lower frequencies, followed by a resonant peak after which the response rapidly decreases. In more detail, the sensitivity in the flat band is 0.23$\cdot 10^{-3}{\;kPa}^{-1}$ for both the thickness-matched design and optical path-matched design, both with a resonance frequency around 13 MHz. Compared to the benchmark, the proposed designs have an enhancement factor with sensitivity of 2.5, while their resonance frequency differs by around 2 MHz. In the following discussion, we analyze the key parameters, including both optical and acoustic sensitivity, to better understand the role of geometrical factors on the various components of sensitivity. Figure 4 shows the results of the optical sensitivity of the two proposed designs.

**Figure 5:**
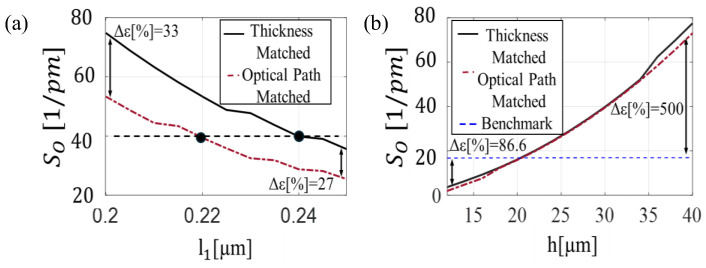
Optical sensitivity for (**a**) different *l*_1_ layer thickness ranging from 0.20 µm to 0.25 µm and (**b**) sensor length *h* ranging from 10 μm to 40 μm.

**Figure 6:**
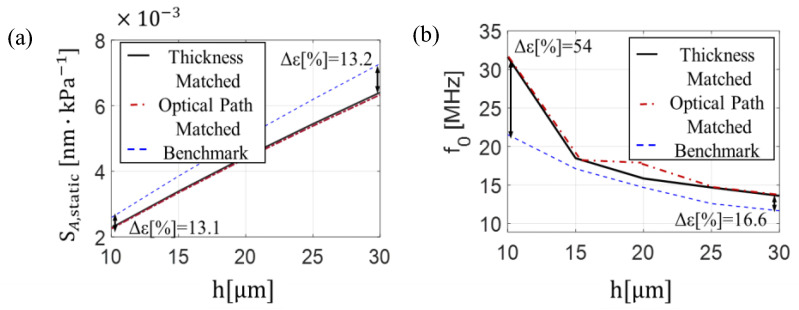
(**a**) Acoustic sensitivity and (**b**) resonant frequency as a function of sensor length of the thickness-matched design (black solid line), optical path-matched design (red dash-dot line), and benchmark configuration (blue dashed line).

From Figure 5a, looking at the optical sensitivity variation as a function of the layer thickness (*l*_1_), the optical sensitivity of the thickness-matched design seems to be higher if compared to optical path-matched design. However, when the layer thicknesses are adjusted to align the reflection spectrum with the *λ_B_*, specifically when *l*_1_ = *l*_2_ = 0.24 μm for the thickness-matched design and *l*_1_ = 0.218 μm and *l*_2_ = 0.26 μm for the optical path-matched design, both designs have the same optical sensitivity value of approximately 40 ${pm}^{-1}$. This result indicates that the optical sensitivity is primarily determined by the operating wavelength rather than by the difference in layer thickness between the two designs. In Figure 5b, we examine the optical sensitivity as a function of the sensor length (h). Here, both designs show very similar behavior, with the sensitivity increasing linearly as the sensor length increases. Specifically, the sensitivity increases with a slope of 25 between sensor lengths of 10 μm and 40 μm, reaching a maximum value at 40 μm of 76 ${pm}^{-1}$. In comparison, the benchmark structure has a sensitivity basically maintained at 15 ${pm}^{-1}$ for different h values. Figure 6 illustrates the results of the acoustic analysis, showing the relationship between sensor length, acoustic sensitivity, and resonance frequency.

As shown in Figure 6a, the mechanical responses of the two proposed designs overlap, highlighting their similar trend. The acoustic sensitivity ($S_{A,static}$) as a function of sensor length exhibits a linear increase with a slope of 0.17, starting from a value of $2.5\cdot 10^{-3}$ [nm$\cdot {kPa}^{-1}$]. However, this slope is slightly lower compared to the benchmark structure, which starts from 2.2 [nm$\cdot {kPa}^{-1}$] and increases with a slope of 0.20. Figure 6b reveals the typical trade-off between sensitivity and bandwidth, showing the variation of the resonance frequency with sensor length. Indeed, as the sensor length increases, a corresponding decrease in the resonance frequency is observed. Notably, when compared to the benchmark configuration, which consists of homogeneous material, the sensitivity and resonance frequency of the multilayer designs are slightly changed. Since the Young’s modulus influences the mechanical response of the structure, this behavior can be attributed to the specific values of Young’s modulus for the materials used in the multilayer configurations, as detailed in Section 2.1. The results obtained through the numerical method further validate and confirm the trends observed in the initial design, reinforcing the accuracy of the modeling approach. Moreover, the similar acoustic behavior observed across the polymer and multilayer structures can be explained by the fact that, despite differences in geometry, the material composition varies only slightly. Additionally, the small layer thicknesses relative to the acoustic wavelength minimize the impact of structural differences, resulting in comparable acoustic responses.

#### 3.2.2. π-Shifted Structure

**Figure 7:**
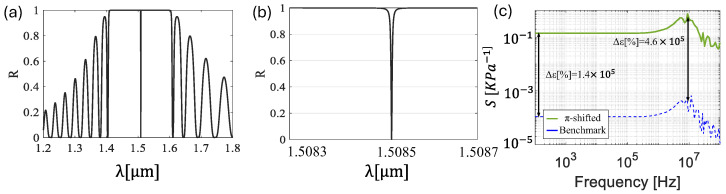
(**a**) Interference spectrum of pi-shifted structure. (**b**) Zoom in on the central dip in the reflection spectrum being analyzed. (**c**) Sensitivity as a function of the frequency at *h* = 30 µm of π-shifted design (green line) and benchmark configuration (blue dashed line).

In this section, we investigate a π-shifted structure, where symmetry breaking is introduced by adding a central layer, $l_{\pi }$. This parameter represents a structural variation equivalent to inserting a thin polymeric region between two highly reflective multilayer stacks. The primary focus of this analysis is on the optical behavior, as the acoustic response remains essentially unchanged compared to previous results. Indeed, since the perturbation in the π-shifted design is much smaller than the acoustic wavelength, it does not significantly affect acoustic performance. Figure 7 presents the key outcomes of the numerical analysis, including the interference spectrum, a zoomed view of the resonance peak emerging from symmetry breaking, and the sensitivity as a function of frequency, all obtained using the numerical model described in Section 2.2.

**Figure 8:**
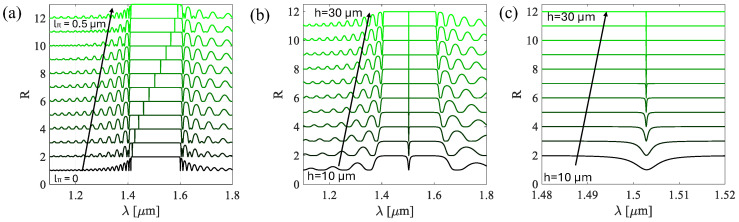
Interference spectra as a function of (**a**) *l_π_* ranging from 0.1 µm to 0.5 µm and (**b**) sensor length *h*. (**c**) Zoom in on the central peak of the reflection spectrum as a function of cavity length. The darker green color indicates lower values of the parameter of interest.

Figure 7a presents the broad reflection spectrum of the π-shifted design, spanning from the first null at *λ* = 1.41 µm to the second null at *λ* = 1.6 µm, excluding the (a) peak located at *λ* = 1.50 µm. The sharp resonance peak is a direct result of this perturbation of *l_π_* = 0.26 µm introduced into the system. As in the previous section, where sensitivity was evaluated around the first peak on the left of the reflectivity maximum, in this case, we focus on this new peak caused by the perturbation. The selected peak is shown in Figure 7b. In Figure 7c, the sensitivity of the π-shifted structure as a function of frequency is reported, exhibiting a flat response at lower frequencies, followed by a resonant response with sensitivity reaching 0.7 kPa^−1^ within the passband, with a resonance frequency centered around 9 MHz close to the benchmark value. The π-shifted design exhibits an improvement in sensitivity, with a two-orders-of-magnitude enhancement compared to the benchmark. These results clearly demonstrate that the π-shifted design significantly enhances the sensor sensitivity, without affecting the peak frequency and bandwidth. We next focus on how changes in design parameters, such as h and *l_π_*, influence the position and shape of the interference peak, thereby impacting the overall optical response of the sensor. This effect is visually reported in Figure 8, where variations in these parameters clearly modify the reflection spectrum.

**Figure 9:**
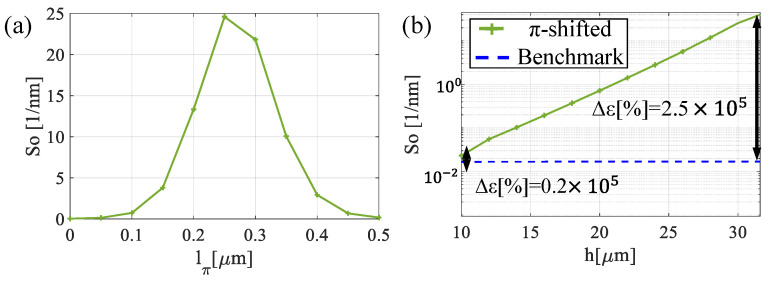
Optical sensitivity as a function (**a**) of the perturbation *l_π_* and (**b**) *h*.

Figure 8a shows how the interference peak reacts to the perturbation variation. As the parameter *l_π_* increases, varying from 0 to 0.5 µm, the wavelength of the peak gradually shifts from *λ* = 1.417 µm to *λ* = 1.59 µm. As shown in Figure 8a, within this range of *l_π_* values, the interference peak can be observed to shift within the broad reflectivity window (whose maximum reflectivity is approximately 1), demonstrating that the spectral dip moves across the entire response bandwidth of the structure. On the other hand, in Figure 8b, the influence of sensor length is considered. Although the position of the peak remains constant at *λ* = 1.50 μm, the sensor length significantly influences the shape of the interference peak, as highlighted in Figure 8c. To further quantify these effects, the optical sensitivity is analyzed as a function of both *l_π_*, and *h* (Figure 9a,b).

Figure 9a confirms that as the perturbation increases, the optical sensitivity increases, reaching a maximum value of 25 [1/nm] at *l_π_* = 0.26 μm, i.e., when the dip appears in the middle of the passband. After this value, the sensitivity decreases, indicating that further increases in *l_π_* have a diminishing effect on the optical response. Moving to Figure 9b, the impact of sensor length h on the optical sensitivity reveals that it increases exponentially with sensor length, reaching a value of 24 [1/nm] at 30 µm. Notably, compared to the benchmark structure, the optical sensitivity shows a significant improvement, with an enhancement factor of three orders of magnitude.

### 3.3. Performance Comparison in Pursuit of a Realistic Implementation

**Figure 10:**
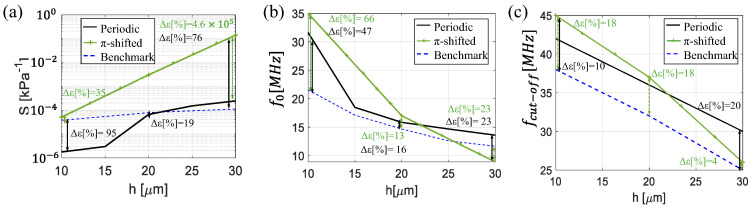
(**a**) Sensitivity, (**b**) peak frequency $f_{0}$, and (**c**) cut-off frequency *f_cut-off_* (−3 dB) of periodic structure (black solid line), π-shifted (green line with marks), and benchmark configuration (blue dashed line).

The performance of all structures in terms of sensitivity (*S*) in the flat band and resonant frequency ($f_{0}$) and cut-off *f_cut_* (−3 dB) is shown in Table 1. In Figure 10, we also present the trends of the two key parameters (*S* and $f_{0}$) across the different designs as a function of sensor length. This allows us to assess the overall performance resulting from the various geometrical configurations. As the optical path-matched and thickness-matched designs exhibit similar trends in both acoustic and optical sensitivity, as well as in their frequency response, we refer to them as a symmetric configuration in the following analysis to avoid redundancy.

Figure 10a shows that all configurations have an increase in sensitivity as *h* increases. For sensor lengths below 20 µm, the periodic multilayer structure performs poorly, likely due to the reduced number of layers resulting from the limited thickness. This compromises the formation of a well-defined reflection spectrum, as insufficient optical interference from multiple interfaces diminishes the effectiveness of the sensing mechanism. However, as the sensor length increases up to 20 µm, the sensitivity improves. At a sensor length of 30 µm, the periodic structure achieves a sensitivity enhancement of 2.5 compared to the benchmark, while the π-shifted configuration further improves performance, reaching a three-orders-of-magnitude sensitivity increase. This highlights the advantage of the π-shifted design, where the introduction of symmetry breaking further enhances sensor sensitivity. Regarding the resonance frequency $f_{0}$, as shown in Figure 10b, it generally decreases as *h* increases, as expected from the trade-off between sensitivity and bandwidth. For the periodic design configuration, the resonance frequency is consistently slightly higher across the range, reaching a maximum value of 32 MHz at 10 µm and a minimum of 13 MHz at 30 µm. Despite providing the highest sensitivity at 30 µm, the π-shifted configuration experiences a decrease in resonance frequency, which in turn also reduces bandwidth compared to periodic configurations. While π-shifted configuration initially at 10 µm exhibits a resonance frequency of 35 MHz with a bandwidth of around 40 MHz, it has a minimum bandwidth of 9 MHz at 30 µm. Finally, Figure 10c illustrates the bandwidth of the different structures. As shown in Figure 10c, the effective detection bandwidth decreases with increasing total thickness h, consistent with the trend observed for the resonance frequency. The π-shifted configuration shows the largest bandwidth at smaller thicknesses (h = 10 μm), where it reaches approximately 45 MHz, but experiences a faster reduction as h increases, reaching about 25 MHz at h = 30 μm. The periodic configuration follows a similar trend but maintains slightly higher bandwidth values over the entire range, whereas the benchmark design remains systematically lower. This behavior confirms the expected trade-off between sensitivity and bandwidth. Overall, the π-shifted design exhibits a dramatically enhanced sensitivity, making it a highly competitive candidate for high-sensitivity applications such as photoacoustic imaging.

#### Dynamic Range and Fabrication Tolerance Considerations

As part of the performance comparison, with a view toward realistic implementation and testing of the proposed structures, we focus on two key aspects: the dynamic operating range and tolerance to fabrication-induced thickness variations. The dynamic range defines the interval over which the sensor response remains linear and reliable, thereby determining the applicability of the device to different signal intensities. Moreover, fabrication tolerance is crucial for assessing robustness in realistic conditions, where small deviations from nominal dimensions are intrinsic in fabrication process. Together, these two factors establish the bridge between ideal design and practical usability.

**Figure 11:**
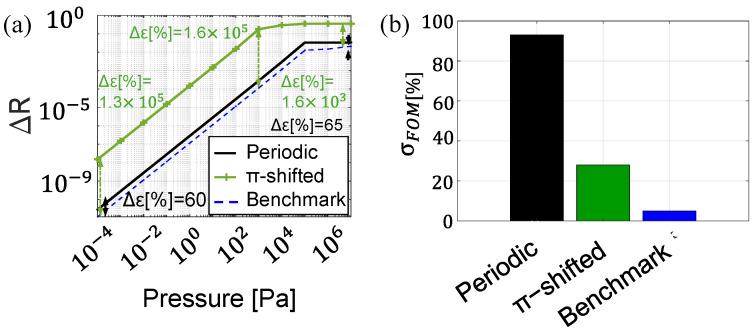
(**a**) Reflectivity variation (ΔR) as a function of incident acoustic wave pressure for the configurations: periodic configuration (black solid line), π-shifted (green line with markers), and benchmark (blue dashed line). (**b**) $\sigma_{FOM}$ [%] of the reflectivity response for each configuration.

The dynamic operating range of the sensor is defined as the frequency interval over which the reflectivity variation remains linear, ensuring reliable signal detection. Figure 11a shows the reflectivity variation ΔR evaluated over a wide span of acoustic pressures from $10^{-4}$ Pa to $10^{7}$ Pa. While the periodic configuration exhibits a dynamic range comparable to that of the benchmark, the π-shifted configuration saturates at $10^{3}$ Pa due to its sharp peak edges, leading to the strongly improved sensitivity. However, this range, within the kilopascal scale, is well suited to applications in ultrasound diagnostics based on photoacoustics, as demonstrated in related studies [2,15].

To take into account the effects of fabrication-induced thickness non-uniformities, we performed a statistical tolerance analysis by introducing random variations of up to ±5% in the individual layer thicknesses. These values align with the inherent variability introduced during the fabrication process, which is influenced by factors such as polymer viscosity, spin speed, and environmental conditions [13,27]. Figure 11b shows the $\sigma_{FOM}$ for each configuration. The statistical analysis of the FOM (see Section 2.3) reveals that the strong sensitivity improvement comes at the cost of reduced robustness to fabrication imperfections when compared to the FP-based benchmark configuration, which exhibits a $\sigma_{FOM}$ of only 4%. Specifically, periodic configuration shows a $\sigma_{FOM}\;value$ exceeding 90%, indicating significant variability in their optical response due to geometrical irregularities. For this configuration, variations in layer thickness can alter the interference pattern, resulting in a less defined or shifted dip, thus demanding extremely precise fabrication to ensure uniform layer thickness and sensor consistency. Even a ±5% tolerance in layer thickness does not provide consistent results, indicating the need for fabrication precision well beyond this level. On the other hand, the interference peak introduced by the asymmetric structure significantly enhances the system robustness against thickness-induced perturbations, resulting in a $\sigma_{FOM}$ of 24%. Hence, the π-shifted configuration can tolerate fabrication-induced dimensional deviations up to ±5% while maintaining a stable response, demonstrating robustness that makes the design suitable for practical implementation. Therefore, the π-shifted design offers an appealing trade-off between sensitivity and fabrication tolerance.

## 4. Conclusions

In this study, we investigated the behaviour of a multilayer structure as an optical fiber based opto-acoustic detector. We proposed both periodic and aperiodic configurations, hereafter referred to as thickness-matched, optical path-matched, and π-shifted designs.

The design rules were first obtained using well-established equations, followed by validation and numerical analysis through an FEM approach. We investigated the effect of mechanical and optical response on the sensitivity *S* and resonance frequency $f_{0}$. This allowed us to understand how structural symmetry and its disruption affect the sensor acoustic and optical response. The results were compared with a planar FP as a benchmark structure.

From a mechanical standpoint, the multilayer structures exhibited similar behavior, with no significant advantages in terms of acoustic performance or bandwidth. Results suggest that the periodic structures are primarily influenced by the operating wavelength, rather than by the differences in layer thickness between the two proposed designs, i.e., thickness-matched and optical-path-matched. This means that using layer thickness based on optical path matched or on layer thickness matched designs yields similar sensitivity performance, with no clear advantage of one criterion over the other. Nonetheless, both periodic approaches allow for a sensitivity increase up to a factor of 2.5 compared to the benchmark and exhibit a comparable wide dynamic operating range. Furthermore, in periodic configuration, a trade-off between performance and manufacturability occurs, underscoring the need for advanced and tightly controlled manufacturing techniques to fully exploit the sensors capabilities. The π-shifted structure, introducing symmetry-break, leads to a significant increase in sensitivity, up to three orders of magnitude higher than the benchmark at the expense of a reduced dynamic operating range. Nevertheless, the resulting bandwidth of about 25 MHz up to kilopascal scale make it suitable for practical applications. This combination of high sensitivity, adequate bandwidth, and practical fabrication tolerance makes the π-shifted design particularly attractive for high-sensitivity and wideband applications, for example, for photoacoustic imaging applications [9]. 

## Figures and Tables

**Table 1:** Sensitivity *S* in the flat band, resonance frequency $f_{0},$ and cut-off frequency *f_cut-off_* (−3 dB) of the proposed structures at *h* = 30 µm.

Configurations	$S\;[{kPa}^{-1}]$	$f_{0}$ [MHz]	$f_{\mathrm{cut-off}}$ (−3 dB) [MHz]
Periodic structure	0.23$\cdot 10^{-3}$	13	30
π-shifted structure	0.15	9	25
Benchmark	0.10$\cdot 10^{-3}$	12	26

## Data Availability

The data supporting the findings of this study are available and can be provided upon reasonable request.
